# G6Pase location in the endoplasmic reticulum: Implications on compartmental analysis of FDG uptake in cancer cells

**DOI:** 10.1038/s41598-019-38973-1

**Published:** 2019-02-26

**Authors:** Mara Scussolini, Matteo Bauckneht, Vanessa Cossu, Silvia Bruno, Anna Maria Orengo, Patrizia Piccioli, Selene Capitanio, Nikola Yosifov, Silvia Ravera, Silvia Morbelli, Michele Piana, Gianmario Sambuceti, Giacomo Caviglia, Cecilia Marini

**Affiliations:** 1 Department of Mathematics (DIMA), University of Genoa, Genoa, Italy; 2 Nuclear Medicine Unit, Department of Health Science, University of Genova, Genoa, Italy; 3 Nuclear Medicine Unit, Policlinico San Martino Hospital, Genoa, Italy; 4 Department of Experimental Medicine, University of Genoa, Genoa, Italy; 5 Unit of Cellular Biology, Policlinico San Martino Hospital, Genova, Italy; 6 Department of Pharmacy, Biochemistry Laboratory, University of Genova, Genova, Italy; 7 CNR Institute SPIN, Genoa, Italy; 8 CNR Institute of Bioimages and Molecular Physiology, Milan, Italy

## Abstract

The favourable kinetics of ^18^F-fluoro-2-deoxyglucose (FDG) permits to depict cancer glucose consumption by a single evaluation of late tracer uptake. This standard procedure relies on the slow radioactivity loss, usually attributed to the limited tumour expression of G6P-phosphatase (G6Pase). However, this classical interpretation intrinsically represents an approximation since, as in all tissues, cancer G6Pase activity is remarkable and is confined to the endoplasmic reticulum (ER), whose lumen must be reached by phosphorylated FDG to explain its hydrolysis and radioactivity release. The present study tested the impact of G6Pase sequestration on the mathematical description of FDG trafficking and handling in cultured cancer cells. Our data show that accounting for tracer access to the ER configures this compartment as the preferential site of FDG accumulation. This is confirmed by the reticular localization of fluorescent FDG analogues. Remarkably enough, reticular accumulation rate of FDG is dependent upon extracellular glucose availability, thus configuring the same ER as a significant determinant of cancer glucose metabolism.

## Introduction

The wide clinical success of PET/CT imaging in cancer largely relies on the accumulation kinetics of ^18^F-fluorodeoxyglucose (FDG) that permits to evaluate the whole body without the need for complex mathematical analysis of tracer blood-tissue exchanges. This clinical standard relies on two key observations: 1) FDG competes with glucose for transmembrane transport and phosphorylation; 2) radioactivity trapped in cells cannot be lost. These two fundamental properties have been formalized by Sokoloff *et al*.^1^ in a seminal paper that so far represents the theoretical basis for the experimental use of ^14^C-2DG and for the clinical value of FDG uptake^2^. The former property has been supported by different studies showing that FDG, like 2-deoxyglucose (2DG), competes with glucose for transmembrane carriers (GLUT)^3,4^ and for hexokinase-catalysed conversion to FDG6P^5–7^. The latter remark has been less clearly illustrated: actually, FDG6P and 2DG6P are false substrates for G6P-isomerase and G6P-dehydrogenase (G6PD), channelling G6P to glycolysis and pentose phosphate shunt, respectively^5,7,8^.

Both substrates can be hydrolysed by G6Pase and thus released into the bloodstream. The assumption at the basis of Sokoloff paper^1^ and of other papers published in the 90s^9–11^ is that in virtually all cancer lesions FDG6P dephosphorylation, though present, is a very slow rate process and can be disregarded at least in the first hour after injection.

**Figure 1:**
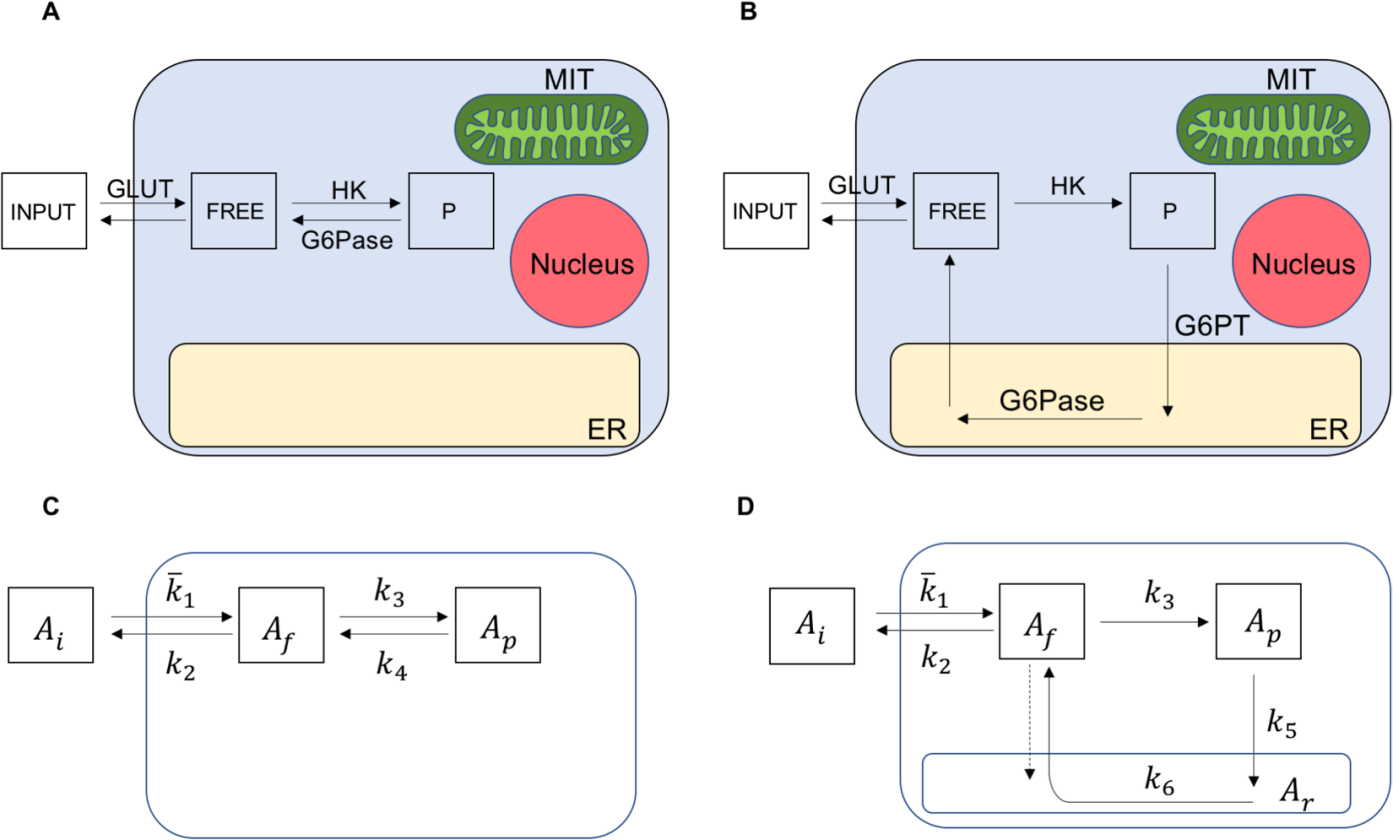
Biochemical models underlying interpretation of cell FDG uptake. (**A**) Conventional model for FDG kinetics considering G6Pase location within the cytosol. (**B**) Proposed model for FDG kinetics considering G6Pase sequestration within the ER lumen and the transport of FDG6P into the ER by G6PT. (**C**,**D**) Compartmental representation in terms of the model activities and rate constants.

However, tracer kinetics can be described by a compartmental model^12^ accounting for three pools which implicitly postulates a free access of FDG6P to the catalytic activity of G6Pase (Fig. 1A,C). Specifically, although modelling analysis actually indicates that dephosphorylation occurs at a very low rate, Graham *et al*.^13^ reported that neglecting its contribution systematically causes an underestimation of glucose consumption rate. Further, a large literature documented that G6Pase is compartmentalized within the lumen of the endoplasmic reticulum (ER)^2,14^. On a first hand, this evidence might explain the accumulation kinetics pattern of FDG despite the high G6Pase activity observed in cancer^15^. On the other hand, the presence of a measurable, though limited, hydrolysis of FDG6P, in virtually all tissues, intrinsically implies a specific mechanism for its transport across the ER membrane. Indeed, FDG release has been found to reflect the expression of the reticular G6P transporter (G6PT) more than that of G6Pase^16^. These remarks lead to the introduction of a fourth ER compartment as an essential feature to model cell FDG retention (Fig. 1B,D).

Our study aimed to verify whether G6Pase sequestration in the ER lumen is compatible with the accumulation kinetics of FDG. To this end, a novel *in vitro* system, able to warrant the needed steady state condition of tracer exchanges, was employed to provide the time activity curves (TACs) of FDG uptake in cultured cells. Two models were applied to the analysis of tracer kinetics for comparison of the results: the standard Sokoloff model and our proposed model accounting for the role of ER (Fig. 1). The results show that ER sequestration of G6Pase is needed to explain cell FDG accumulation.

## Results

### Instrument calibration

**Figure 2:**
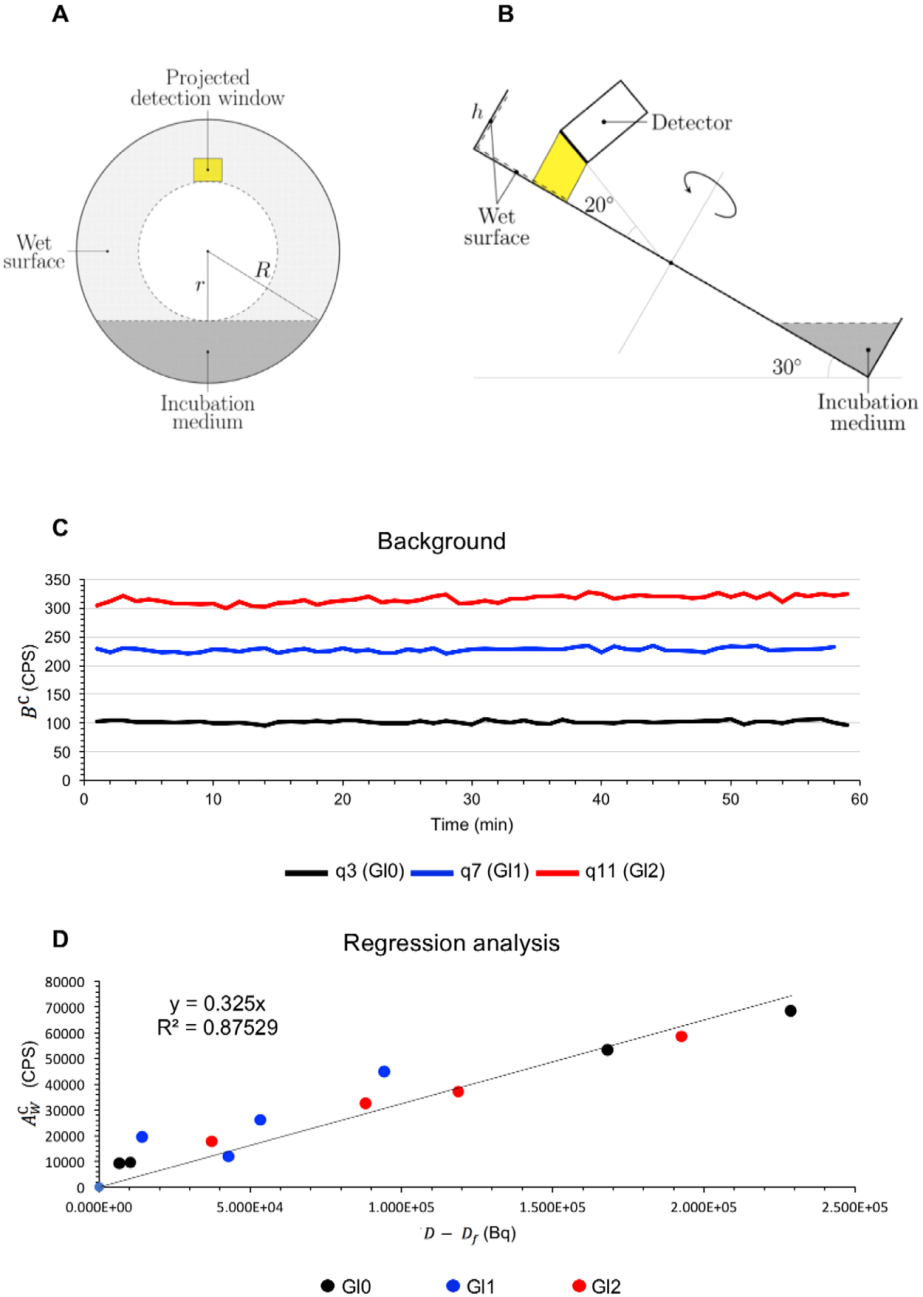
Calibration of LigandTracer measurements. (**A**) Pictorial representation of the front view of the LT device: Petri dish radii r = 23 mm, R = 43.5 mm; incubation medium occupying the wet circular segment of central angle 2cos^−1^(r/R); projected detection window as yellow area whose radioactivity is collected by the LT detector. (**B**) Pictorial representation of the lateral view of the LT device, where the height of the wet lateral surface is h = 11.85 mm. (**C**) TACs of the measured background *B*^*C*^ (CPS) for three selected cell-free experiments: q3 (Gl0) at zero glucose (black line), q7 (Gl1) at glucose concentration 5.5 mM (blue line), and q11 (Gl2) at glucose concentration 11.1 mM (red line). (**D**) Simple linear regression on the data couples ($D-{D}_{f},{A}_{W}^{C}$) for the twelve cell-free experiments, made at glucose concentration zero (black points), 5.5 mM (blue points) and 11.1 mM (red points).

FDG kinetics in cells cultured over a Petri dish (PD) was evaluated using a dedicated instrument, LigandTracer White (Uppsala Se) (LT)^17–19^. As schematized in Fig. 2A,B, LT allows positioning of the PD on a plate periodically rotating around an axis inclined at a 30° angle from the vertical. An electron/positron detector faces the zenith of plate orbit, while the incubation medium is obviously limited to its lowest part and thus does not contaminate the counting rate of cultured cells. At each cycle (minute) *t*, the detector measures the counting rates (in counts per second, CPS) of background (*B*^C^) and target cells through the whole 180 minutes experiment duration.

Our first step aimed to estimate the calibration factor needed to convert the measured CPS in activity (Bq) and thus to write dimensionally consistent equations. To this purpose, 12 vials containing four mL of incubation medium with different glucose concentration (0 mM, n = 4; 5.5 mM, n = 4; 11.1 mM, n = 4) were enriched with variable amounts of FDG. Three mL of this radioactive fluid, containing an amount *D* (Bq) of radioactivity, were spilled in the LT-lodged PD and measurements were performed for 60 minutes. At the end of the procedure, the medium was accurately collected and its radioactivity content *D*_*f *_(Bq) was measured. The decay-corrected difference in fluid radioactivity *D* − *D*_*f*_ was thus regarded as the radioactivity contaminating the PD.

As shown in Fig. 2C, the measured counting rate (*B*^C^) reached the equilibrium at its maximum value within the first minute and remained stable thereafter in all instances. As described in the Methods, this rapid diffusion permitted us to estimate the fraction *D* − *D*_*f*_ eventually exposed to positron detector, and the corresponding expected counting rate ${A}_{W}^{{\rm{C}}}$ (in CPS) generated by emission from the PD wet surfaces and estimated through *B*^C^ (equation (11)). Regression analysis (Fig. 2D) of the whole experimental data set of 12 samples (Table 1) showed a tight linear correlation between *D* − *D*_*f*_ and ${A}_{W}^{{\rm{C}}}$, regardless either glucose or radioactivity concentrations in the spilled fluid. The slope of this line was thus considered the efficiency coefficient *e* (0.3 CPS/Bq, SEE 2.6 × 10^−2^ CPS/Bq).

**Table 1:** Experimental values for all cell-free experiments, indexed as q1, …, q12: glucose concentration (mM), administered FDG activity *D* (MBq), final FDG activity *D*_*f*_ (MBq), end-time value of the wet activity ${A}_{W}^{C}$ (CPS), and percentage of FDG removal off the medium (computed as the difference *D* − *D*_*f*_, over *D*).

	Gl (mM)	*D* (MBq)	*D*_*f*_ (MBq)	${A}_{W}^{C}$ (CPS)	%removal
q1	0	6.79	6.78	1.57 × 10^4^	0.11
q2	0	5.17	4.95	6.45 × 10^4^	4.42
q3	0	4.41	4.40	9.63 × 10^3^	0.18
q4	0	4.25	4.08	5.33 × 10^4^	3.96
q5	5.5	5.83	5.74	4.49 × 10^4^	1.62
q6	5.5	4.40	4.35	3.31 × 10^4^	1.21
q7	5.5	3.01	2.99	2.33 × 10^4^	0.48
q8	5.5	1.49	1.44	1.18 × 10^4^	2.90
q9	11.1	6.56	6.37	5.84 × 10^4^	2.94
q10	11.1	4.67	4.55	3.71 × 10^4^	2.54
q11	11.1	3.93	3.84	3.24 × 10^4^	2.25
q12	11.1	2.15	2.11	1.76 × 10^4^	1.75

This analysis provided us with the method to convert the measured counting rates in time curves of activities and thus to estimate the rate constants of exchanges between the different pools, in analogy with the conventional analysis of time concentration curves^1,4,7–11^. In fact, the analysis reported in Supplementary Material S1 indicates that this approach provides a unique set of rate constants for each experiment^20^ and thus ensures that the reconstructed numerical values of kinetic parameters are the only ones explaining the data^21,22^. We also observe that the estimated rate constants are comparable with the standard ones, with the only exception of FDG rate of entry into the cells (*k*_1_) that has to be converted into its counterpart (${\bar{k}}_{1}$) accounting for the ratio between intracellular (*V*_*cyt*_) and medium (*V*_*i*_) volume, according to the equation:

1 $${\bar{k}}_{1}={k}_{1}\frac{{V}_{cyt}}{{V}_{i}}.$$

### Uptake experiments

FDG uptake kinetics was evaluated in the murine breast cancer model of 4T1 cells seeded in standard PDs according to the procedures described in the Method section. Incubation medium was enriched with a known amount of FDG (≅2 MBq/mL) and with two different glucose concentrations: 5.5 mM (1 g/L, n = 4, Gl1) and 11.1 mM (2 g/L, n = 4, Gl2).

**Figure 3:**
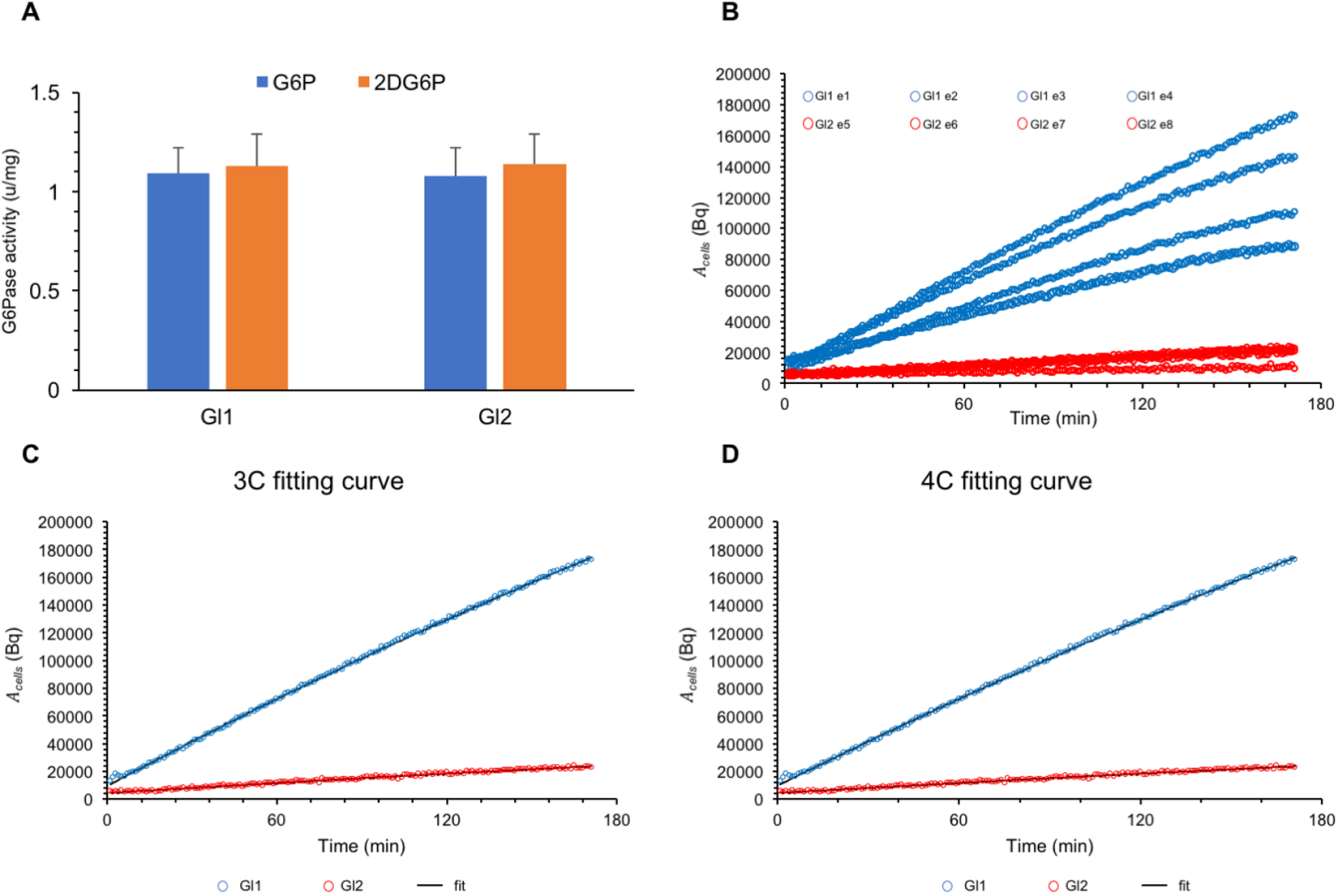
FDG uptake by cultured cells. (**A**) G6Pase activity of Gl1 and Gl2 cell lysates. No difference could be observed for catalytic rate on G6P (blue) and 2DG6P (orange). Similarly, enzyme function was not affected by extracellular glucose concentration. (**B**) TACs of FDG inside cells (*A*_*cells*_) for all the experiments at 5.5 mM (Gl1, blue lines) and 11.1 mM (Gl2, red lines). (**C**,**D**) Example of model fitting curve as the adherence of the predicted curve (black solid line) to selected Gl1 (blue dotted lines) and Gl2 (red dotted lines) experimental data, for the 3C and 4C designs, respectively. The black fitting line is very difficult to visualize, since the predicted curve completely overlaps the experimental dots.

Experimental conditions are reported in Table 2: the average cell number was 488 ± 213 × 10^3^ (Gl1) and 513 ± 232 × 10^3^ (Gl2) (p = ns); the administered FDG dose *D* was 6.87 ± 1.24 (Gl1) and 4.76 ± 0.34 MBq (Gl2) (p = ns). In agreement with our previous observation, G6Pase activity of studied 4T1 cells approached values previously reported in rat liver homogenates^15^. Similarly, the rate of catalysis was superimposable for both G6P and 2DG6P (1.09 ± 0.13 U/mg vs 1.13 ± 0.16 U/mg, respectively, p = ns), as shown in Fig. 3A.

**Table 2:** Experimental values for all the cell experiments, indexed as e1, …, e8: glucose concentration (mM), number of cells, administered FDG activity *D* (MBq), and end-time value of the TAC of FDG inside cells (*A*_*cells*_ at 180 min).

	Gl (mM)	cell number	*D* (MBq)	*Acells*(180)
e1	5.5	8.00 × 10^5^	8.37	1.73 × 10^5^
e2	5.5	4.50 × 10^5^	6.00	1.46 × 10^5^
e3	5.5	5.00 × 10^5^	5.34	1.11 × 10^5^
e4	5.5	2.00 × 10^5^	7.75	8.88 × 10^4^
e5	11.1	3.50 × 10^5^	5.22	2.29 × 10^4^
e6	11.1	3.00 × 10^5^	4.73	2.13 × 10^4^
e7	11.1	8.00 × 10^5^	4.4	2.03 × 10^4^
e8	11.1	6.00 × 10^5^	4.7	1.10 × 10^4^

According to Fig. 3B, the time course of FDG uptake was almost linear in all cell cultures, with small random oscillations related to experimental errors of counting statistics. The noticeable difference between the curves of the same group is obviously dependent on the product initial dose and number of cells present in each experiment.

Compartmental analysis was performed according to both the ER accounting model (4 compartments, 4C) and the standard Sokoloff design (3 compartments, 3C)^1^. As detailed in  Fig. 1, the 4C configuration extends the conventional 3C and subdivides intracellular FDG6P pool into two compartments: one accounting for cytosol, and one for ER pool, with the latter representing an additional source for the free FDG, besides the extracellular input compartment. In other words, 4C assumes that hydrolysis of FDG6P must be preceded by its transport into the ER lumen, with a rate constant indexed as *k*_5_, while the restitution rate of FDG6P to the free FDG by G6Pase is indexed as *k*_6_. Free FDG flux from cytosol to ER is disregarded, assuming an early equilibrium warranted by the recognized wide abundance of GLUT carriers in the ER membrane^23^.

Both models provided an excellent fit between reconstructed curves and experimental TACs, with relative error being <6% (1.3 ± 0.4% at 5.5 mM, and 5.3 ± 0.2% at 11.1 mM) in all measured times in all experiments (Fig. 3C,D). In particular, this analysis proved that the accumulation kinetics of FDG observed in cancer cells is compatible with ER sequestration of G6Pase.

Estimated values of the rate constants and their variability among the experiments of the two conditions (Gl1 vs Gl2) are reported for both 3C and 4C in Table 3. Actually, both models provided similar estimates for transmembrane transport both to (${\bar{k}}_{1}$) and from (*k*_2_) cultured cells. By contrast, accounting for the ER compartment increased almost six-fold the value of *k*_3_ and thus the estimate of FDG phosphorylation rate constant.

**Table 3:** Rate constants (min^−1^) of the 3C and 4C models for all the cell experiments, indexed as e1, …, e8, made at 5.5 mM (Gl1) and 11.1 mM (Gl2), with *e* = 0.3 CPS/Bq.

	3C				4C				
	${\bar{k}}_{1}$	*k*_2_	*k*_3_	*k*_4_	${\bar{k}}_{1}$	*k*_2_	*k*_3_	*k*_5_	*k*_6_
**Gl1**									
e1	0.0060	4.9255	0.1080	0.0013	0.0055	4.4798	0.7192	4.4563	0.0015
e2	0.0081	4.5348	0.0994	0.0026	0.0077	4.2792	0.6508	4.6374	0.0030
e3	0.0047	3.7491	0.1226	0.0027	0.0041	3.2282	0.8643	3.5131	0.0033
e4	0.0069	4.3444	0.0503	0.0033	0.0070	4.4844	0.3176	3.4072	0.0035
**Mean**	**0.0064**	**4.3885**	**0.0951**	**0.0025**	**0.0061**	**4.1179**	**0.6380**	**4.0035**	**0.0028**
**SD**	**0.0014**	**0.4901**	**0.0313**	**0.0008**	**0.0016**	**0.6008**	**0.2314**	**0.6332**	**0.0009**
**Gl2**									
e5	0.0037	4.0619	0.0267	0.0012	0.0043	4.8096	0.1657	2.5317	0.0012
e6	0.0050	3.7189	0.0169	0.0014	0.0050	3.7482	0.1031	2.0301	0.0014
e7	0.0059	4.2664	0.0152	0.0011	0.0067	4.8754	0.0913	3.1222	0.0011
e8	0.0033	2.5509	0.0051	0.0003	0.0033	2.5782	0.0304	1.7209	0.0003
**Mean**	**0.0045**	**3.6495**	**0.0160**	**0.0010**	**0.0048**	**4.0029**	**0.0976**	**2.3512**	**0.0010**
**SD**	**0.0012**	**0.7665**	**0.0088**	**0.0005**	**0.0014**	**1.0812**	**0.0554**	**0.6130**	**0.0005**

### Reticular location of G6Pase and intracellular exchanges of phosphorylated hexoses

The metabolic response to glucose availability in the incubation medium was differently reconstructed by the two compartmental designs. In the 3C model, shifting from Gl1 to Gl2 induced a moderate decrease in ${\bar{k}}_{1}\,$and *k*_2_, a large reduction in *k*_3_ and a significant decrease in *k*_4_ (Table 3). Switching to the 4C model, obviously provided a relatively more detailed picture. Although responses of free FDG (${\bar{k}}_{1}$, *k*_2_ and *k*_3_) reproduced the readout of 3C, description of FDG6P fate was markedly different. In fact, doubling glucose concentration virtually halved the rate constant of ER radioactivity accumulation (*k*_5_) and dephosphorylation-release (*k*_6_) (Table 3).

**Figure 4:**
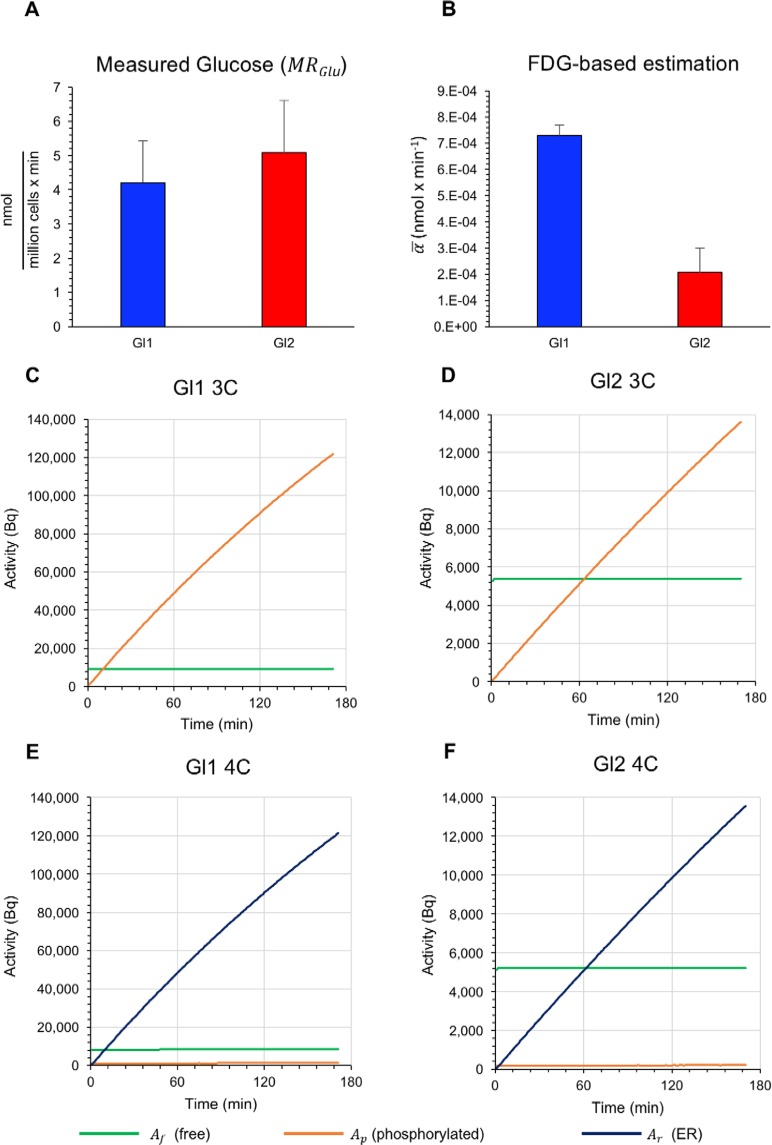
Glucose consumption and time activity curves of FDG inside model compartments. (**A**) Comparable glucose consumption in Gl1 (blue) and Gl2 (red) experiments measured directly. (**B**) Glucose consumption as estimated by compartmental analysis on FDG uptake, showing a marked decrease in the estimated index of glucose intake from Gl1 to Gl2 experiments. (**C**,**D**) TACs of the model compartments for the 3C model, for both Gl1 and Gl2 experiments. (**E**,**F**) TACs of the model compartments for the 4C model, for both Gl1 and Gl2 experiments. Each panel (**C**,**D**,**E**,**F**) reports the mean value curves of the TACs computed over the experiments of the same group, with *e* = 0.3 CPS/Bq; the green, orange, and purple curves refer to the free (*A*_*f*_), cytosolic-phosphorylated (*A*_*p*_), and ER-phosphorylated (*A*_*r*_) activities (Bq), respectively. Note that the label of the vertical axes of the Gl1 figures (**C**,**E**) and of the Gl2 figures (**D**,**F**) refers to two different orders of magnitude (10^4^ vs 10^5^).

A similar difference between 3C and 4C also applied to the trend of reconstructed compartment activities. In the 3C model, shifting from Gl1 to Gl2 experiments almost halved intracellular abundance of free FDG (Fig. 4C,D). However, the reduction of *k*_3_ caused an even more evident decrease in FDG6P accumulation, as documented by the ratio between FDG6P and FDG content *A*_*p*_/*A*_*f*_ that decreased six-fold (from 13.3 ± 1.2 to 2.5 ± 0.4). 4C estimates of free FDG were remarkably similar with respect to the conventional model. However, FDG6P content became dependent upon time, due to a progressive radioactivity transfer to the ER compartment (*A*_*r*_). Actually, cytosolic FDG6P radioactivity *A*_*p*_ was 15% of FDG content in Gl1 experiments and decreased down to 4% in Gl2 cultures (Fig. 4E,F). Finally, the most evident response was related to the transfer of intracellular FDG6P toward the ER since the ratio *A*_*r*_/(*A*_*f*_ + *A*_*p*_) decreased from 14 ± 1.3 in Gl1 to 2.4 ± 0.5 in Gl2 (p < 0.001, Fig. 4E,F).

### Reticular location of G6Pase and Lumped Constant interpretation

FDG accumulation kinetics is usually reconstructed to estimate glucose consumption rate (*MR*_*Glu*_). However, GLUT transport system as well as hexokinase and G6Pase catalysis can display different affinities for glucose and FDG. These differences require normalization through a lumped constant (*LC*) in order to estimate *MR*_*Glu*_ by FDG uptake analysis^9,10,13,24^. The procedure has been described since the introduction of 2DG method by Sokoloff *et al*.^1^. As detailed in Supplementary Material S2, conventional estimation of *MR*_*Glu*_ can be computed according to the following expression:

2 $$M{R}_{Glu}=\frac{1}{LC}\frac{{k}_{1}{k}_{3}}{{k}_{2}+\,{k}_{3}}{C}_{glu},$$

where *C*_*glu*_ is the concentration of glucose in the medium (in Supplementary Material S2 it has been denoted as ${C}_{i}^{g}$). Shifting measurements from concentrations to activities asks to consider equation (1) and thus to rewrite the conventional formulation of equation (2) as follows:

3 $$M{R}_{Glu}LC=\frac{{k}_{1}{k}_{3}}{{k}_{2}+{k}_{3}}{C}_{glu}=\frac{{V}_{i}}{{V}_{cyt}}\frac{{\bar{k}}_{1}{k}_{3}}{{k}_{2}+{k}_{3}}{C}_{glu}.$$

To simplify the previous expression (equation (3)), it is convenient to introduce the quantity $\bar{\alpha }$ defined as

4 $$\frac{{\bar{k}}_{1}{k}_{3}}{{k}_{2}+{k}_{3}}{C}_{glu}=\bar{\alpha },$$

so that the following equation is obtained

5 $$M{R}_{Glu}LC=\frac{{V}_{i}}{{V}_{cyt}}\bar{\alpha }.$$

The coefficient $\bar{\alpha }\,$(equation (4)) can be evaluated by using also the estimated rate constants, or the graphical analysis proposed by Patlak *et al*.^25^ directly applied to the TACs.

In our experimental setting, glucose removal from the incubation medium, i.e. true *MR*_*Glu*_, was similar in Gl2 and Gl1 experiments (5.2 ± 1.3 nmol × min^−1^ vs 4.2 ± 1.1 nmol × min^−1^, respectively, p = ns; Fig. 4A). However, using both 3C and 4C compartment models, halving glucose availability (from Gl2 to Gl1) increased $\bar{\alpha }$ from 0.21 ± 0.08 nmol × min^−1^ to 0.74 ± 0.20 nmol × min^−1^, p < 0.01 (Fig. 4B).

Since the cells were cultured under identical conditions, and the different Gl1 and Gl2 glucose concentrations in the medium were used only during the experiment, by applying equation (5) we evaluated the ratio

6 $$\frac{M{R}_{Glu1}LC}{M{R}_{Glu2}LC}=\,\frac{{V}_{i}/{V}_{cyt}\,{\bar{\alpha }}_{1}}{{V}_{i}/{V}_{cyt}\,{\bar{\alpha }}_{2}},$$

where the subscripts 1 and 2 refer systematically to Gl1 and Gl2, respectively; the constants *LC* and *V*_*i*_/*V*_*cyt*_ in equation (6) can be simplified leading to

7 $$\frac{M{R}_{Glu1}\,}{M{R}_{Glu2}}=\frac{\,{\bar{\alpha }}_{1}}{\,{\bar{\alpha }}_{2}}.$$

The left-hand side of equation (7) was evaluated by substitution of the true values of *MR*_*Glu*_, while the right-hand side was computed by replacement of the FDG-estimated values for the coefficients $\bar{\alpha }$. The resulting estimates were

$$\frac{M{R}_{Glu1}\,}{M{R}_{Glu2}}\cong 0.8,$$

and

$$\frac{{\bar{\alpha }}_{1}}{{\bar{\alpha }}_{2}}\cong \mathrm{3.5.}$$

This largely different response of true *MR*_*Glu*_ with respect to its FDG-based estimation, consistent with Fig. 4A,B, implies that halving glucose availability causes an immediate increase in *LC*. As a consequence, *LC* cannot be regarded as constant, but its value depends upon the extracellular glucose concentration, in agreement with the data previously reported by Noll *et al*.^11^.

In the framework of the 3C model, the only possible explanation for this mismatch is a change in transmembrane transport system or hexokinase asset. However, this interpretation seems relatively unlikely, due to the sudden nature of observed response. By contrast, it is at least partially explained by the 4C model. As detailed in Supplementary Material S2, the metabolic rate of glucose for the 4C model can be written as

8 $$M{R}_{Glu}={k}_{5}\frac{{C}_{p}}{{C}_{i}}\frac{{C}_{glu}}{LC},$$

where *C*_*p*_ = *A*_*p*_/*V*_*cyt*_ remains constant early after FDG exposure (Fig. 4). We found that doubling glucose concentration virtually halves *k*_5_ and markedly decreases *A*_*p*_. Therefore, the combination in equation (8) indicates a marked deceleration of FDG6P transport to ER and seems consistent with a selective decrease in ER accumulation of radioactivity, able to explain the marked decrease in *LC*.

### Imaging confirmation

**Figure 5:**
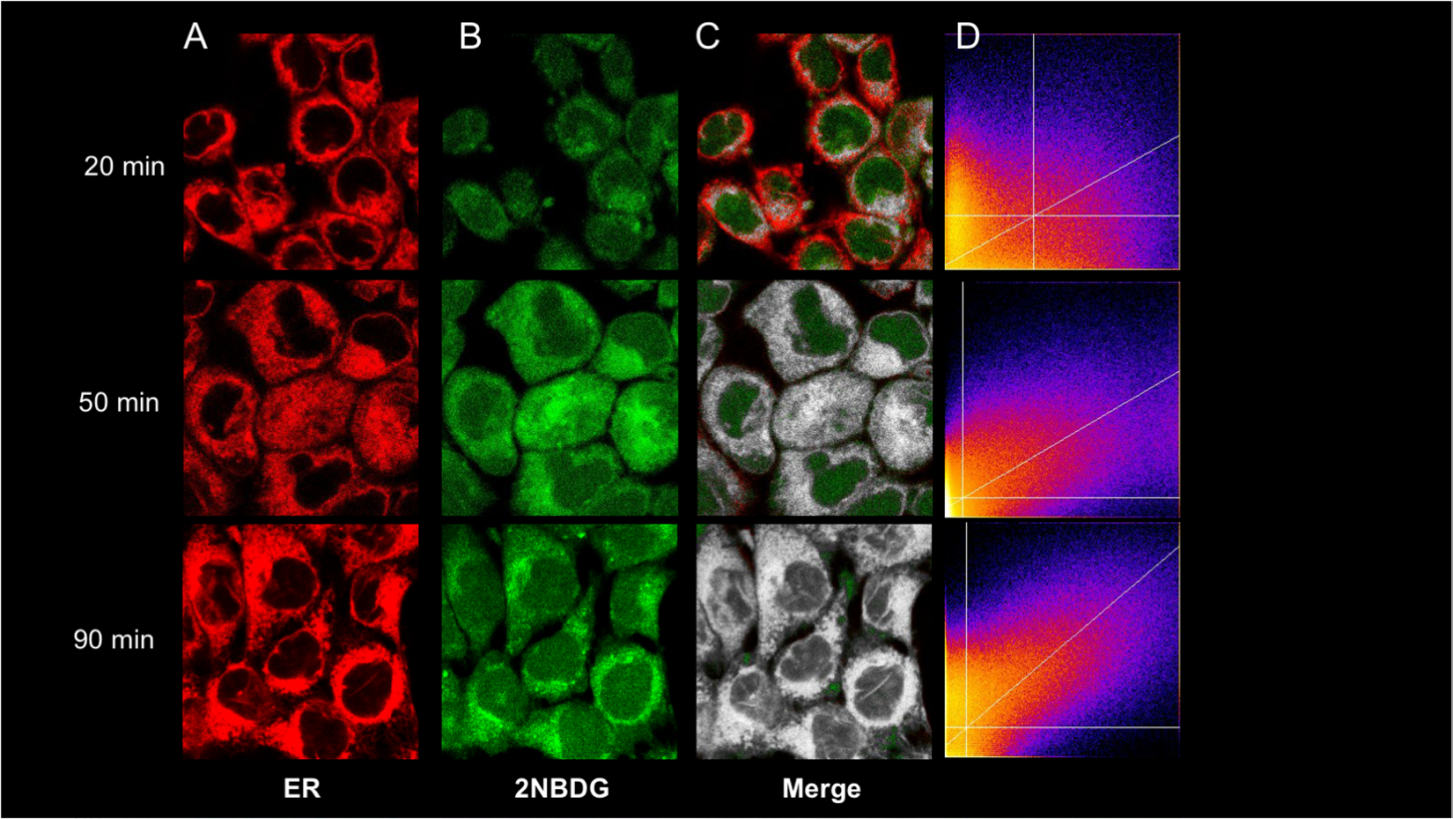
Imaging confirmation of radioactivity accumulation in ER. (**A**) Images obtained with the vital ER staining glybenclamide. (**B**) Simultaneous distribution of the fluorescent 2DG analogue 2NBDG. Images are obtained at 20, 50 and 90 minutes, as reported. (**C**) Colocalization (in white) of ER and 2NBDG signal at a spatial resolution of 250 nm. (**D**) Costes representation of the same images.

Accounting for the documented sequestration of G6Pase in the ER shifted the compartmental description of intracellular FDG kinetics, configuring the same ER as the radioactivity accumulation site. To overcome the limited spatial resolution of radionuclide detection and to corroborate this theoretical finding, we thus extended our study by verifying whether this ER fate also applies to the fluorescent 2DG analogue 2-NBDG. To this purpose, three cell cultures were exposed to a solution containing glibenclamide as a vital ER probe, as well as glucose and 2-NBDG at the concentration of 5.5 mM and 50 µM, respectively. Incubation lasted 20, 50 or 90 minutes before imaging with confocal microscopy with a spatial resolution of 400 nm. Images were analysed using a dedicated routine of ImageJ and proved a progressive increase in the colocalization between the hexose fluorescence and ER signal (Fig. 5).

## Discussion

To the best of our knowledge, this study represents the first characterization of FDG kinetics in cultured cancer cells. As expected, the reconstructed time curves of activities following from application of the standard Sokoloff model^1^ fairly fit the data, but the extremely low rate of radioactivity loss disagrees with the high cell G6Pase activity. This mismatch may come from a limited access of FDG6P to the hydrolysing enzyme, due to the luminal localization of G6Pase in the ER^2,14^. Therefore, existence of the ER pool for FDG6P has been considered; the mathematical analysis of data has confirmed this choice by the identification of a continuous tracer transport from the cytosol to the ER, a markedly faster rate of hexokinase-catalysed reaction, and accumulation of FDG6P in the ER. The last result has also been supported by the independent evidence of a progressive ER accrual of the fluorescent FDG analogue 2-NBDG. Thus, the switch from the conventional 3C to the 4C model has provided a valuable alternative description of FDG kinetics.

In detail, the standard Sokoloff design implies that FDG retention can be regulated only by transmembrane transport and hexose phosphorylation. As a consequence, the combined study of GLUT function and hexokinase activity (or isoforms expression) has been almost invariably adopted to explain variations in *LC*^9,10,13,24^. Accounting for the acknowledged reticular sequestration of G6Pase deeply modifies the mathematical interpretation of tracer uptake mechanisms, and provides a possible explanation for the largely conflicting findings so far provided by the conventional approach. This point is explained by looking at the readout of 3C and 4C response to doubling competition by glucose molecules in the medium. Applying both models, the shift from Gl1 to Gl2 slightly decreases the rate of transmembrane transport as well as the rate of phosphorylation. In the 3C model, this effect eventually reduces FDG6P accumulation. In the 4C design, cytosolic FDG6P activity remains far lower than free FDG and modestly contributes to overall cell radioactivity content. However, an increase in glucose availability causes a profound reduction in ER accumulation, since the marked decrease in the estimated content of cytosolic FDG6P is combined with an almost halving of *k*_5_. On a practical ground, this observation is of limited relevance, since the spatial resolution of positron detection is insensitive to intracellular tracer location. However, it has deep theoretical implications since it indicates that G6P competes with FDG6P for G6PT mediated transport from cytosol to ER.

The main features of FDG kinetics resulting from the introduction of the ER pool may be summarized in two points: (1) ER significantly contributes to G6P metabolism, and (2) ER contribution to G6P processing is inversely related to extracellular glucose availability, thus explaining the previously reported evidence of an inverse relationship between *LC* and glucose availability^1,11,26^.

Standard compartmental analysis deals with time concentration curves in the different pools to the aim of retrieving the rate constants of tracer exchanges. Our procedure modified this approach by writing an equivalent system in terms of activities, regarded as the state variables replacing concentrations. The change was motivated by several considerations: (1) source data were represented by a cell counting rate that was measured in CPS and directly converted in activity (Bq) through the efficiency factor; (2) evaluation of tracer concentration would have required an estimation of the total intracellular volume, whose accuracy would have been inevitably hampered by the unknown number of cell sources of radioactivity; (3) as detailed in Supplementary Material S1, analysing TACs instead of time concentration curves did not affect values of rate constants describing intracellular exchanges, provided that the adimensional ratio *v* = *V*_*er*_/*V*_*cyt*_ (i.e. the ratio between ER and cytosol volumes) could be considered independent of total number of cells. Relying on previous studies in hepatocytes, the ratio *v* was set at 0.17. Indeed, regardless its numerical value, the ratio was reasonably considered stable over the three hours’ duration of the experiment, allowing a reliable estimate of the rates coefficients.

Obviously, despite an apparent reliability of the efficiency coefficient *e*, conversion of counting rates to activity might have been affected by a number of errors, including inaccurate estimates of initial and final medium activities, non-uniform FDG distribution on the wet surface, non-orthogonal emission from the surface under the detector. Accordingly, a test on the effects of variations of *e* on the reconstructed rate constants was carried out. Specifically, each experiment was analysed considering three different *e* values, namely, the chosen value, corresponding to 1/*e* = 3, 1/*e* = 1.8 as minimum, and 1/*e* = 4.2 as maximum, thus accounting for a variability up to 40%. Increasing or decreasing *e* value actually modified significantly ${\bar{k}}_{1}$ (50%) for both the 3C and 4C models. The high variability of ${\bar{k}}_{1}$ with respect to alteration of the *e* value was inherently dependent on modelling assumptions, as shown in Supplementary Material S3. On the contrary, the uncertainty of the efficiency coefficient *e* did not affect significantly the estimated values of rate constants associated with intracellular processes: for the 3C model, 10% of variability for *k*_2_ was found, down to <4% for *k*_3_ and *k*_4_; similarly, for the 4C model, 10% of variability for *k*_2_, down to <4% for *k*_3_ and *k*_6_, and 15% for *k*_5_. On these grounds, we considered the reconstructed rate constants values as sufficiently stable, despite the relative uncertainty in the estimate of *e*.

The analysis of TACs provided an estimate of the transmembrane tracer exchange rate ${\bar{k}}_{1}$ markedly lower than *in vivo* estimates of *k*_1_^27–31^. This apparent mismatch simply reflected the fact that ${\bar{k}}_{1}$ described the tracer exchange between cytosolic and incubation volumes, with *V*_*cyt*_<<*V*_*i*_. On the other hand, both ${\bar{k}}_{1}\,$and *k*_2_ were found to be independent of the model chosen for the reduction, indicating that the model design did not affect the mathematical description of transmembrane tracer flux from the medium to the cytosol and vice versa.

FDG6P hydrolysis was estimated to a similar rate by either *k*_4_ or *k*_6_ for the 3C or 4C model, respectively. In agreement with previous literature, reviewed in Table 2 of Reivich *et al*.^32^, the numerical values of *k*_4_ and *k*_6_ were very small (in the order of 10^−3^), suggesting a modest contribution of this process to radioactivity trend. Yet, FDG hydrolysis was measurable and its presence led to a deep change in the theoretical description of radioactivity fate within a cell. This remark suggests that the a priori constraint *k*_4_ = 0 might be a relatively inaccurate procedure, as already observed by Graham *et al*.^13^.

Regarding *k*_3_ estimates, data obtained with 3C model nicely agreed with previous *in vivo* literature^30–32^ as opposed to the 4C estimates that set this rate constant to markedly higher values. Thus the difference is related to the model design: unlike the standard assumption, 4C considers a transfer of phosphorylated hexose to the ER. The resulting continuous removal of FDG6P from the cytosol preserves the gradient between FDG6P and FDG concentrations. This gradient justifies the estimated high rate of hexokinase-catalysed phosphorylation, which is actually close to the rate predicted on the basis of computational modelling of enzyme function estimated by the conventional Michaelis-Menten descriptors (Supplementary Material S4).

Besides the already mentioned methodological assumptions, several biochemical considerations have to be carefully discussed to correctly interpret the general consequences of our results. As a first point, despite the possible general nature of our model, FDG kinetics was only evaluated in cancer cells. Actually, the limited FDG uptake in unstimulated normal cells would have hampered TACs analysis.

As a second consideration, the high abundance of GLUT receptors in ER membranes^23^ justifies the release of free FDG from the ER lumen to cytosol, but inevitably implies a transfer in the opposite direction. The consequent direct FDG transfer from cytosol to ER was not considered in 4C and, thus, the present analysis does not provide any determination of non-phosphorylated FDG amount within the ER. However, the ER lumen and cytosol can be considered as rapidly exchanging pools reaching relative equilibrium in a rather short time, unless a processing machine active on large amounts of non-phosphorylated hexoses is hypothesized in the former compartment.

The basis of our mathematical analysis, i.e. the G6Pase localization in the ER lumen, was not directly verified. Nevertheless, this interpretation was proposed a long time ago and has been virtually confirmed by all studies investigating the regulation of glucose release in the bloodstream^14^. Moreover, the high G6Pase activity reported for both glucose and 2DG is incompatible with a free access of FDG6P to the hydrolytic reaction in the cytosol. Similarly, the carrier transferring phosphorylated hexoses across the ER membrane was not clearly identified. However, a large literature showed the existence of a dedicated ATP-dependent G6PT. Again, the universally recognized evidence of a measurable G6Pase function in virtually all studied tissues intrinsically implies the presence of this carrier and its capability to recognize FDG6P as a substrate.

Finally, the present study did not evaluate whether radioactivity release from labelled cells reflected a true FDG loss or, rather, the consequence of radiolysis, and thus the loss of ^18^F^−^ ions. This task was not feasible because of the relatively low number of FDG molecules eventually escaping tagged cells. However, this point has been reported for the same 4T1 cells, since thin layer chromatography showed that virtually all activity present in the medium was accounted by free FDG^15^.

In conclusion, the present study shows that the acknowledged location of G6Pase function in the ER lumen is compatible with FDG kinetics in cultured cancer cells, and deeply modifies our current interpretation of the relationship between cell FDG uptake and glucose intake. Accounting for enzyme sequestration implies an almost six-fold higher rate of hexokinase catalytic rate. It also implies a continuous transfer of FDG6P from the cytosol to ER. The rate of this process is inversely related to the extracellular glucose concentration, and thus indicates a relevant access of G6P to G6PT. Despite a high FDG6P concentration combined with a high G6Pase activity in the ER lumen, FDG6P hydrolysis is remarkably slow. This disagreement corroborates the concept of a reticular metabolic machinery able to process this G6P analogue, and competing with G6Pase to prevent its function^15,33^. The nature of this metabolism, its regulating enzymes, and factors able to modulate its rate cannot be defined in the present study. However, should it be confirmed, ER accumulation might configure a new significance for FDG uptake, far beyond its current role of accessible index of glucose consumption.

## Methods

### LigandTracer Calibration

As schematized in Fig. 2A,B, LT encompasses a periodically rotating plate with an electron/positron detector facing the orbit zenith, and incubation medium limited to its nadir. For this set of experiments, each rotation cycle was divided into 4 intervals: (a) 25 seconds with cell culture in the rotation nadir and hence fully immersed in the radioactive medium; (b) 5 seconds for a counter-clockwise rotation of 180°; (c) 25 seconds with cell culture in the rotation zenith and thus under the detector; (d) 5 seconds for counter-clockwise rotation of 180°, leading to cycle restart. At each cycle (minute) *t*, the detector measures collected counts in phases (a) and (c) in order to estimate the counting rates (CPS) of background (*B*^C^), and target cells (*T*^C^), respectively. The decay corrected difference

9 $${A}_{{\rm{LT}}}^{{\rm{C}}}={T}^{{\rm{C}}}-{B}^{{\rm{C}}}$$

is regarded as the time course of the counting rate of radiation emitted by cells through the whole 180 minutes of the experiment duration.

Consider now cell-free PDs. The LT may be regarded as a closed system in which the administered radioactive dose *D* is exhaustively distributed in the medium, on the wet surface of the circular ring, and the lateral surface of the cylinder of height *h* (Fig. 2B). Elementary geometric considerations (Fig. 2A,B) lead to evaluation of the total emitting wet area *S*_*W*_ as:

$${S}_{W}\approx 7.5\times {10}^{3}{{\rm{mm}}}^{2}.$$

The area *S*_*W*_ represents the background pool and can be reasonably considered invariant, since the same PDs and the same medium volume have been used for all experiments. It was also found that the measured counting rate (*B*^C^) reached the equilibrium at its maximum value within the first minute and remained stable thereafter for all experiments (Fig. 2C). Moreover, a further series of four experiments, in which counting was performed every 90°, showed that the distribution of sources on the emitting surface could be regarded as homogeneous. Positrons received by the detector were regarded as coming from the orthogonal projection of the detection window over the PD surface. The detection window of the LT is rectangular in shape with an area of 80 mm^2^ (8 × 10 mm); however, since it points toward the plate with an angle of 20° with respect to the normal, its projection on the PD is a surface of area

$${S}_{pdw}\approx 75\,{{\rm{mm}}}^{2}.$$

The time stability of *B*^C^ counting rate (in CPS) together with homogeneity led to the proportionality law

10 $$\frac{{A}_{W}^{{\rm{C}}}}{{S}_{W}}=\frac{{B}^{{\rm{C}}}}{{S}_{pdw}},$$

or, equivalently,

11 $${A}_{W}^{{\rm{C}}}=\frac{{S}_{W}}{{S}_{pdw}}{B}^{{\rm{C}}}\approx 100\,{B}^{{\rm{C}}},$$

where ${A}_{W}^{{\rm{C}}}$ represented the expected counting rate (in CPS) associated with the dose *D* − *D*_*f*_ (in Bq) contaminating the wet surface *S*_*W*_ of the PD. The conservation law of activity for the closed LT system was thus expressed by the linear equation:

12 $${A}_{W}^{{\rm{C}}}=e\,(D-{D}_{f}),$$

where for each experiment the counting rate ${A}_{W}^{{\rm{C}}}$ were related to *B*^C^ as in equation (11). Equation (12) defined the ‘efficiency coefficient’ *e* (CPS/Bq), for the conversion of Bq to measured CPSs and vice versa, as the slope of the regression line determined by the experimental set (Fig. 2D).

### Cultured cells

Murine 4T1 breast cancer cells were cultured in standard PDs with 100 mm diameter, inclined at 30° from the horizontal plane so as to limit cell presence to the lowest segment of the circular ring. Once seeded, cells were maintained in DMEM medium with glucose concentration set at 11.1 mM (2 g/L) enriched with 10% fetal bovine serum. For each experiment, a pair of twin cultures was prepared for cell counting and for radioactivity measurements, respectively. To this purpose, the PD was placed on the platform of the LigandTracer White (Uppsala Se) instrument^17–19^ schematized in Fig. 2A,B. Cultures were washed with PBS before the administration of 3 mL of incubation medium. For all experiments, FDG concentration ranged from 1 to 2 MBq/mL. By contrast, glucose concentration was set at 5.5 mM (1 g/L, Gl1 n = 4) or 11.1 mM (2 g/L, Gl2 n = 4), respectively. At the end of the procedure, G6Pase activity was measured at 660 nm, following the inorganic phosphate production according to the Fiske and Subbarow method.

### Time activity curve in cultured cells and input function

TACs of cell cultures were reconstructed from LT data assuming both a homogeneous metabolic pattern and a uniform distribution of cells over the surface covered by the incubation medium, represented by a circular segment *c*, described in Fig. 2A, of area *S*_*c*_ ≈ 1.1 · 10^3^ mm^2^. To obtain the estimated TAC of the whole cell culture, denoted as *A*_*cells*_, the LT-measured CPS, namely ${A}_{{\rm{LT}}}^{{\rm{C}}}$ (equation (9)), were normalized according to the following equation:

13 $${A}_{cells}=\frac{1}{e}\frac{{S}_{c}}{{S}_{pdw}}{A}_{{\rm{LT}}}^{{\rm{C}}},$$

where the constant

$$\frac{1}{e}\frac{{S}_{c}}{{S}_{pdw}}$$

was fixed for all experiments at 42 Bq/CPS.

Next, the conservation law of activity was applied to the LT, regarded as closed-system, in order to describe the time course of tracer activity in the incubation medium, and thus the input function, in terms of *A*_*cells*_ (equation (13)). The activity administered at the beginning of an experiment was denoted by *D*. This activity remained in the medium for less than one minute, due to the instantaneous sequestration of the activity ${A}_{W}={A}_{W}^{{\rm{C}}}/e$ on the wet PD surface. Thus, the amount of tracer activity actually available for cell uptake at the beginning of the experiment was set as

14 $${A}_{i0}=D-{A}_{W}.$$

As a consequence of conservation, the tracer contained in the medium at time t (i.e. the input function) was determined as the difference:

15 $${A}_{i}(t)={A}_{i0}-{A}_{cells}(t).$$

### Mathematical model

The standard approach to compartmental analysis^12,34,35^ is based on consideration of time concentration curves. Within the LT framework, we found convenient to reformulate the mathematical problem in terms of TACs, with the caveats and consequences described in Supplementary Material S1. For the 4C model described in Fig. 1B,D, a system of three linear first-order Ordinary Differential Equations (ODEs) with constant rate coefficients *k* (min^−1^) was obtained. The system expresses the rate of change of the compartmental activities *A*_*f*_, free cytosolic, *A*_*p*_, phosphorylated cytosolic, and ${\bar{A}}_{r},$redefined phosphorylated reticular, as

16 $${\dot{A}}_{f}=-\,({k}_{2}+{k}_{3}){A}_{f}+{k}_{6}{\bar{A}}_{r}+{\bar{k}}_{1}{A}_{i}$$

17 $${\dot{A}}_{p}={k}_{3}\,{A}_{f}-{k}_{5}{A}_{p}$$

18 $${\dot{\bar{A}}}_{r}={k}_{5}{A}_{p}-{k}_{6}{\bar{A}}_{r}$$

where

19 $${\bar{k}}_{1}={k}_{1}\frac{{V}_{cyt}}{{V}_{i}},\,{\bar{A}}_{r}={A}_{r}\frac{{V}_{cyt}}{{V}_{er}};$$

the superposed dot denotes the time derivative, and explicit reference to time dependence is usually omitted. The initial conditions are ${A}_{f}(0)={A}_{p}(0)={\bar{A}}_{r}(0)=0$. Here *A*_*i*_ is the given input function (equation (15)). The redefined ER activity ${\bar{A}}_{r}$ is related to the “true” activity *A*_*r*_ through the dimensionless ratio *V*_*cyt*_/*V*_*er*_ (equation (19)), where *V*_*cyt*_ is the total volume of cytosol in the cell culture, and *V*_*er*_ is the corresponding volume of the ER. In the definition of ${\bar{k}}_{1}$ (equation (19)), *V*_*i*_ is the volume of the liquid medium, and *k*_1_ is the usual rate constant of the formulation in terms of concentrations; the same interpretation as rate constants for concentrations holds for the coefficients *k*_2_, *k*_3_, *k*_5_, and *k*_6_ (see Supplementary Material S1 for details).

The system of ODEs for the activities *A*_*f*_ and *A*_*p*_ of the 3C model (Fig. 1A,C) was written as

20 $${\dot{A}}_{f}=-\,({k}_{2}+{k}_{3}){A}_{f}+{k}_{4}{A}_{p}+{\bar{k}}_{1}{A}_{i}$$

21 $${\dot{A}}_{p}={k}_{3}\,{A}_{f}-{k}_{4}{A}_{p}$$

with initial conditions *A*_*f*_(0) = *A*_*p*_(0) = 0 and given input function *A*_*i*_. Here too we set ${\bar{k}}_{1}={k}_{1}\frac{{V}_{cyt}}{{V}_{i}}$. The rate coefficients *k*_1_, *k*_2_, *k*_3_, and *k*_4_ were also pertinent to the description in concentrations.

### Comparison with data

In the present framework, the *direct problem* is the evaluation of the compartment activities for any given set of rate constants: for the 4C model, this means solving the system of equations (16), (17), (18), given ***z***_5_ = (${\bar{k}}_{1}$, *k*_2_, *k*_3_, *k*_5_, *k*_6_); for the 3C model, solving equations (20), (21), given ***z***_4_ = (${\bar{k}}_{1}$, *k*_2_, *k*_3_, *k*_4_). The related *inverse problem* consists in the determination of ***z***_5_ or ***z***_4_ for any given input function *A*_*i*_(*t*) (equation (15)) and its corresponding total cellular activity *A*_*cells*_(*t*) (equation (13)). Here, the total activity *A*_*cells*_ of cultured cells is set equal to the sum of the compartment activities.

In the case of the 4C model, the equation connecting the datum *A*_*cells*_ to *A*_*f*_, *A*_*p*_, and *A*_*r*_ is written as

22 $${A}_{cells}=\,{A}_{f}+{A}_{p}+{A}_{r}={A}_{f}+{A}_{p}+v\,{\bar{A}}_{r},$$

where *v* = *V*_*er*_/*V*_*cyt*_. Indeed, *v* is independent of the number of cells and coincides with the ratio of the intracellular volumes of ER and cytosol. According to previous studies in hepatocytes^36^, *v* was set at 0.17. In the equation connecting the datum *A*_*cells*_ with the model-compartment activities, the activities depend on the unknown vector of parameters ***z***_5_; consequently, that equation represents the starting point for the formulation and the solution of the compartmental inverse problem. Notice that ${A}_{r}=v\,{\bar{A}}_{r}$ may be explicitly determined after reconstruction of ${\bar{A}}_{r}$.

The same procedure holds also for the 3C model, leading to the equation

23 $${A}_{cells}={A}_{f}+{A}_{p}$$

for the unknown vector of parameters ***z***_4._

It may be shown that the vectors of parameters ***z***_5_ and ***z***_4_ can be uniquely determined by the given data (see^20^). The uniqueness property ensures that the numerical values of kinetic parameters obtained by solving the compartmental inverse problem are the only values explaining the data^21,22^. We recall that the compartmental inverse problems of equation (22) and equation (23) were solved by means of a Newton-type iterative algorithm^37,38^, already used and validated in other works of our group^39–41^.

## Supplementary information

Supplementary Material

## Data Availability

The datasets generated during and/or analysed during the current study are available from the corresponding author on reasonable request.
