# Supplementary material for: G6Pase location in the endoplasmic reticulum: Implications on compartmental analysis of FDG uptake in cancer cells
Source: Sci Rep. 2019 Feb 26;9:2794. doi: 10.1038/s41598-019-38973-1 (PMC6391477; doi:10.1038/s41598-019-38973-1)
Supplement: Supplementary file 1 — Supplementary Material [file 41598_2019_38973_MOESM1_ESM.pdf]

Supplementary Material

G6Pase location in the endoplasmic reticulum: Implications on compartmental analysis of FDG uptake in cancer cells

Mara Scussolini[*1], Matteo Bauckneht[2], Vanessa Cossu[3], Silvia Bruno[4], Anna Maria Orengo[3], Patrizia Piccioli[5], Selene Capitanio[3], Nikola Yosifov[3], Silvia Ravera[6], Silvia Morbelli[2,3], Michele Piana[1,7], Gianmario Sambuceti[2,3,8], Giacomo Caviglia[1], Cecilia Marini[2,3,8]

[1]Department of Mathematics (DIMA), University of Genoa, Genoa, Italy

[2]Nuclear Medicine Unit, Department of Health Science, University of Genova, Genoa, Italy.

[3]Nuclear Medicine Unit, Policlinico San Martino Hospital, Genoa, Italy

[4]Department of Experimental Medicine, University of Genoa, Genoa, Italy

[5]Unit of Cellular Biology, Policlinico San Martino Hospital, Genova, Italy

[6]Department of Pharmacy, Biochemistry Laboratory, University of Genova, Genova, Italy

[7]CNR Institute SPIN, Genoa, Italy

[8]CNR Institute of Bioimages and Molecular Physiology, Milan, Italy

First and Corresponding author:

Mara Scussolini, M.S.
PhD student
Department of Mathematics (DIMA), University of Genoa,
Via Dodecaneso 35, 16146 Genova, Italy

E-mail: scussolini@dima.unige.it

Phone: +39-010-3536644

Supplementary Material S1. Mathematical formulation of compartmental models.

In standard compartmental analysis framework [S1,S2,S3], the flow of tracer, resulting from interchange of radioactive molecules between the model compartments, is described by a system of Ordinary Differential Equations (ODEs) for concentrations with constant coefficients, called rate constants or kinetic parameters. By the use of a LigandTracer (LT) device we obtain time-dependent curves for total tracer activity in cells. In order to derive the system of ODEs in terms of activities, we start with the formulation in terms of concentrations (Bq/mL) denoted by the letter C with a low subscript identifying the compartment.

The system of ODEs for the tracer concentrations C_f, C_p, C_r of the 4C model is written as

$$\dot{C}_f = -(k_2 + k_3)C_f + k_6 C_r + k_1 C_i \quad \text{(S1)}$$

$$\dot{C}_p = k_3 C_f - k_5 C_p \quad \text{(S2)}$$

$$\dot{C}_r = k_5 C_p - k_6 C_r \quad \text{(S3)}$$

with the initial conditions $C_f(0) = C_p(0) = C_r(0) = 0$. The function C_i is the concentration of tracer in the medium. Concentrations and corresponding activities are related by

$$C_f = \frac{A_f}{V_{cyt}}, \quad C_p = \frac{A_p}{V_{cyt}}, \quad C_r = \frac{A_r}{V_{er}}, \quad C_i = \frac{A_i}{V_i}, \quad \text{(S4)}$$

where V_{cyt} is the total volume of cytosol, V_{er} is the total volume of the endoplasmic reticulum (ER), and V_i is the volume of the liquid medium. Substitution of activities in Eqs (S1), (S2), (S3), and multiplication of both sides of the equations by V_{cyt}, lead to the following system of ODEs for the 4C activities:

$$\dot{A}_f = -(k_2 + k_3)A_f + k_6 \bar{A}_r + \bar{k}_1 A_i \quad \text{(S5)}$$

$$\dot{A}_p = k_3 A_f - k_5 A_p \quad \text{(S6)}$$

$$\dot{\bar{A}}_r = k_5 A_p - k_6 \bar{A}_r \quad \text{(S7)}$$

with

$$\bar{k}_1 = k_1 \frac{V_{\text{cyt}}}{V_i}, \quad \bar{A}_r = A_r \frac{V_{\text{cyt}}}{V_{\text{er}}}, \quad \text{(S8)}$$

and initial conditions $A_f(0) = A_p(0) = \bar{A}_r(0) = 0$. The activity A_i of tracer in the medium is the given input function, while $\bar{A}_r$ is an auxiliary unknown. With the only exception of k_1, the two systems of Eqs

(S1), (S2), (S3) and (S5), (S6), (S7) depend on the same rate constants.

Concerning the 3C model, the system of ODEs for the tracer concentrations C_f and C_p is

$$\dot{C}_f = -(k_2 + k_3)C_f + k_4 C_p + k_1 C_i \tag{S9}$$

$$\dot{C}_p = k_3 C_f - k_4 C_p \; . \tag{S10}$$

Straightforward repetition of the above procedure leads to the following system of ODEs for the 3C activities A_f, A_p:

$$\dot{A}_f = -(k_2 + k_3)A_f + k_4 A_p + \bar{k}_1 A_i \tag{S11}$$

$$\dot{A}_p = k_3 \, A_f - k_4 A_p \; . \tag{S12}$$

Intracellular tracer is contained in cytosol and ER. Following a standard approach [S3], we express the total concentration of tracer for the cell culture C_{cells} as

$$C_{cells} = (1 - v_r)(C_f + C_p) + v_r C_r \; , \tag{S13}$$

where $v_r = \frac{V_{er}}{V_{cyt}+V_{er}}$ is the volume fraction of ER with respect to the total volume occupied by tracer.

The total concentration of the cell culture and the related activity A_{cells} obey the equation

$$C_{cells} = \frac{A_{cells}}{V_{cyt}+V_{er}} \; . \tag{S14}$$

Substitution into Eq. (S13) of A_{cells} and replacement of compartment concentrations with the corresponding activities, lead to the equation

$$A_{cells} = A_f + A_p + A_r = A_f + A_p + v \, \bar{A}_r \; , \tag{S15}$$

where the adimensional constant $v = V_{\mathrm{er}}/V_{\mathrm{cyt}}$ is introduced to relate the datum A_{cells} to the solutions of the system of Eqs (S5), (S6), (S7). In the case of the 3C model, Eq. (S13) simplifies to

$$C_{cells} = C_f + C_p \; , \tag{S16}$$

and thus

$$A_{cells} = A_f + A_p \; . \tag{S17}$$

Eqs (S15) and (S17) are applied in this work to connect the available data to the model activities.

Supplementary Material S2. Lumped Constant.

There are well known similarities in the kinetics of FDG and glucose. The Lumped Constant (LC) was introduced to take profit of these similarities in order to estimate the metabolic rate of glucose in terms of the corresponding metabolic rate of FDG. Here we analyse the LC in the framework of the 4C model. To this aim, the definition of LC is first revisited for the standard 3C model, commonly referred to as the Sokoloff model. We adopt a simplified approach with respect to, e.g. [S4] and followers, which however is sufficient for our purposes.

First we examine the flux of glucose from medium to cells, followed by phosphorylation inside cells, under the assumption that glucose kinetics is ruled by the 3C model [S4]. Accordingly, we adopt the notations and symbols already introduced for FDG kinetics, with addition of a superscript g to indicate that they refer to glucose; for example, the concentration of glucose in the medium is denoted by C_i^g. We assume that the flux is stationary, which means that C_i^g and C_f^g are considered constant. We add the condition $k_4^g = 0$, which is the mathematical counterpart of the assumption of a low activity level of G6Pase.

We consider tracer kinetics in the 3C system, described by Eqs (S9), (S10). We assume that: 1) $k_4 = 0$, and 2) tracer flow has reached a stationary state, corresponding to constancy of C_i and C_f. We denote by U_{FDG} and MR_{Glu} the uptake rate of FDG and the metabolic rate of glucose, respectively. These rates are identified with the corresponding net rates of phosphorylation [S2] in the reactions catalysed by hexokinase. Since $k_4 = k_4^g = 0$, we obtain

$$U_{FDG} = k_3 C_f \ , \quad MR_{Glu} = k_3^g C_f^g \ . \tag{S18}$$

Introduction into Eqs (S9), (S10) of the stationary condition yields

$$U_{FDG} = k_3 C_f = \alpha C_i \ , \quad \alpha = \frac{k_1 k_3}{k_2 + k_3} \ . \tag{S19}$$

Eq. (S19) shows that the metabolic rate U_{FDG} is related to the input concentration C_i through the constant factor α; the coefficient α represents the slope of the well known “Patlak plot” [S4] and may be regarded as the rate constant for tracer uptake by the cell system. Indeed, evaluation of the time derivative of Eq. (S16) shows that $\dot{C}_{cells} = \dot{C}_p = k_3 C_f = \alpha C_i$.

In order to connect the metabolic rates of FDG and glucose, we refer to [S4] to write

$$\frac{U_{FDG}}{MR_{Glu}} = \frac{V_m K_m^g C_f}{V_m^g K_m C_f^g} = \frac{k_3 C_f}{k_3^g C_f^g} , \tag{S20}$$

where V_m and K_m are the Michaelis-Menten constants for the phosphorylation reaction; specifically, V_m is the maximum rate of the reaction, while K_m is the concentration C_f that produces a reaction rate of one half the maximum value. We recall explicitly that C_f and C_f^g are constant, and hence also the metabolic rates are constant. Multiplication of both sides of Eq. (S20) by C_i^g / C_i provides

$$\frac{U_{FDG}/C_i}{MR_{Glu}/C_i^g} = LC , \tag{S21}$$

where the Lumped Constant LC is represented as

$$LC = \frac{V_m K_m^g C_f / C_i}{V_m^g K_m C_f^g / C_i^g} = \frac{k_3 C_f / C_i}{k_3^g C_f^g / C_i^g} . \tag{S22}$$

The first expression of LC is consistent with the literature [S2,S4]. It follows from Eq. (S21) that

$$MR_{Glu} = \frac{1}{LC} \frac{U_{FDG}}{C_i} C_i^g . \tag{S23}$$

According to Eq. (S19), we find

$$MR_{Glu} = \frac{1}{LC} \frac{k_1 k_3}{k_2 + k_3} C_i^g , \tag{S24}$$

which provides the required metabolic rate of glucose in terms of the lumped constant LC, the constant α for FDG, and the concentration of glucose in the medium C_i^g. In terms of $\bar{k}_1$, Eq. (S24) may also be written as

$$MR_{Glu} = \frac{1}{LC} \frac{V_i}{V_{cyt}} \frac{\bar{k}_1 k_3}{k_2 + k_3} C_i^g . \tag{S25}$$

Suppose now that: 1) tracer kinetics is described by the 4C model, 2) the (asymptotic) condition of stationarity holds, 3) $k_6 = 0$. According to stationarity C_i, C_f, and C_p are constant, which means that the concentration of each compartment has reached equilibrium, with the exception of ER, where accumulation occurs. As to C_f and C_p, the condition of stationarity is attained in a few minutes, following the related activities; as to C_i, its (approximate) constancy is related to the very limited amount of tracer absorbed by the cell cultures, versus the total content of the medium. The constraint $k_6 = 0$ follows from the remark that k_6 is of order 10^{-3}, and hence it is approximated by zero; it plays the same role as the condition $k_4 = 0$, for 3C model.

As in the case of 3C model, the net rate of phosphorylation of FDG is given by $k_3 C_f = U_{FDG}$, so that Eq. (S18) still holds. Note that, unlike the previous case, $\dot{C}_p = 0$ because of stationarity; however the contribution $-k_5 C_p$, entering Eq. (2), corresponds to flow of phosphorylated tracer towards ER, so that it is not directly involved in the phosphorylation-dephosphorylation process, although it contributes to the rate of concentration C_p in cytosol. As a consequence of Eq. (S1) and stationarity, Eq. (S19) holds and the previous analysis applies. In particular, Eqs (S24) and (S25) provide MR_{Glu}, but the rate coefficients are determined by reduction of the 4C model.

To comment on the procedure, we observe that, strangely enough, the rate constant k_5 does not contribute explicitly to the metabolic rate of FDG, although the the quantity $k_5 C_p$ describes flow from the pool of phosphorylated tracer in cytosol to that of phosphorylated tracer in ER. However, Eqs (S2) and (S3) imply that $k_5 C_p = k_3 C_f = \dot{C}_r$. Therefore, the net phosphorylation rate of FDG may also be written as

$$U_{FDG} = k_5 C_p \quad \text{(S26)}$$

and, since C_p is constant, k_5 may be considered as directly proportional to U_{FDG}. Finally, the metabolic rate of glucose as defined in Eq. (S23) can be rewritten for the 4C model as

$$MR_{Glu} = \frac{1}{LC}\frac{k_5 C_p}{C_i} C_i^g \ . \quad \text{(S27)}$$

Supplementary Material S3. Direct dependence of $\bar{k}_1$ on the efficiency coefficient e.

Simulations at different values of the efficiency coefficient e, accounting for a variability up to 40%, show that the standard deviation for $\bar{k}_1$ is about 50% for both Gl1 and Gl2 data, and reduction by both 3C and 4C models, whereas the other rate coefficients have been left almost unaltered. It is the aim of this section to put forward a qualitative argument showing that the high variability of $\bar{k}_1$ is inherently dependent on modelling assumptions and the system response. For definiteness the 4C model is examined.

We consider the system of ODEs for the activities (S5), (S6), (S7) and discard the contribution $k_6 \bar{A}_r$. This simplification is consistent with the estimates of the rate constants, showing that k_6 is of the order of 10^{-3}. Next we consider an asymptotic condition where the activities of the input, free, and cytosolic phosphorylated compartments assume almost constant values. These assumptions are consistent with our results on tracer kinetics. It follows from Eq. (S7) that $\dot{\bar{A}}_r = k_5 A_p$ is constant. Eqs (S5) and (S6) imply that

$$\dot{\bar{A}}_r = \frac{k_3}{k_2+k_3} \bar{k}_1 A_i \, . \tag{S28}$$

Next we consider the asymptotic expression of the time derivative of Eq. (S15), which reduces to $\dot{A}_{cells} = v \, \dot{\bar{A}}_r$, where both $\dot{A}_{cells}$ and $\dot{\bar{A}}_r$ are constant. Combination of this result with Eq. (S28) leads to

$$\bar{k}_1 = \frac{k_2+k_3}{k_3} \frac{\dot{A}_{cells}}{v} \frac{1}{A_i} , \tag{S29}$$

with v a given physiologic parameter.

We consider dependence on e of $\bar{k}_1$, as given by Eq. (S29). The activity A_i is approximated by $A_{i0} = D - A_W^C/e$, while the time rate $\dot{A}_{cells}$ is replaced by $\dot{A}_{cells}^C/e$. It follows that Eq. (S29) is equivalent to

$$\bar{k}_1 = \frac{k_2+k_3}{k_3} \frac{\dot{A}_{cells}^C}{v} \frac{1}{De-A_W^C} = \frac{\Lambda}{De-A_W^C} , \tag{S30}$$

with Λ, D, A_W^C constant quantities. In particular, Λ is defined in Eq. (S30). We assume that the rate constants k_2 and k_3 are independent of e; this condition is consistent with the results obtained by the simulations performed with varying e. Then, according to Eq. (S30), growth of e implies necessarily decrease of $\bar{k}_1$, and conversely.

Supplementary Material S4. Estimate of k_3 from Michaelis-Menten kinetic constants.

We discuss here an estimate of k_3 which is obtained by comparison with the phosphorylation rate described by means of the Michaelis-Menten law, with values of the constants given by [S5].

The value of the Michaelis-Menten constant K_m for hexokinase II with respect to FDG as substrate is recovered from Table 2 in [S5] as K_m = (174 $\pm$ 15) μM; V_m is reconstructed as V_m = (2.6 $\pm$ 0.30) μM/s, since Table 2 provided the Vmax ratio of FGD with glucose. The constant values are reported also in [S6], Table 1 p.130, but without indication of errors.

The values of K_m and V_m are first transformed in Bq/mL and Bq/mL 1/min, respectively, assuming an order of magnitude of 10^{31}. Then they are substituted into the nonlinear expression of the reaction rate R of phosphorylation which, following from application of the Michaelis-Menten equation, can be written as

$$R = \frac{V_m}{C_f+K_m} C_f \, .$$

The ratio $V_m/(C_f + K_m)$ is identified with k_3. The concentration $C_f = A_f/V_{cyt}$ is estimated as $\cong 44 \cdot 10^7$ Bq, corresponding to $A_f \cong 10^4$ Bq and $V_{cyt} \cong 0.134 \cdot 10^{-3}$ cm^3 for $6 \cdot 10^5$ cells. In particular, C_f is much smaller than K_m so that k_3 reduces to the ratio V_m/K_m. The result is $k_3 = 0.90 \pm 0.13$ 1/min, which is comparable with the mean value of k_3 for the 4C model, Gl1 experiments, shown in Table 3.

Supplementary References

[S1] Carson, R. E. Tracer Kinetic Modeling in PET in *Positron Emission Tomography: Basic Sciences* (ed. Valk, P. E., Bailey, D. L., Townsend, D. W., Maisey, M. N.) 147-179 (Springer, 2005).

[S2] Cherry, S. R., Sorenson, J. A. & Phelps, M. E. *Physics in nuclear medicine* (Elsevier, 2012).

[S3] Wernick, M. N. & Aarsvold, J. N. *Emission Tomography: The Fundamentals of PET and SPECT* (Academic Press, 2004).

[S4] Sokoloff, L., Reivich, M., Des Rosiers, M. H., Patlak, C. S., Pettrigrew, K. D., Sakurada, O. & Shinihara, M. The [14C]deoxyglucose method for the measurement of local cerebral glucose utilization: theory, procedure, and normal values in the conscious and anesthetized albino rat. *J. Neurochem*. **28**, 897-916 (1977).

[S5] Muzi, M., Freeman, S. D., Burrows, R. C., Wiseman, R. W., Link, J. M., Krohn, K. A., Graham, M. M. & Spence, A. M. Kinetic characterization of hexokinase isoenzymes from glioma cells: implications for FDG imaging of human brain tumors. *Nucl. Med. Biol.* **28**, 107-116 (2001).

[S6] Wang, Q., Liu, Z., Ziegler, S. I. & Shi, K. A Reaction-Diffusion Simulation Model of [^{18}F]FDG PET Imaging for the Quantitative Interpretation of Tumor Glucose Metabolism in *Computational methods for molecular imaging* (ed. Gao, F., Shi, K. & Li, S.) 123-137 (Springer, 2015).
